# Access to genetic evaluation of 1463 individuals with orofacial cleft in Brazil

**DOI:** 10.1016/j.jped.2024.07.002

**Published:** 2024-07-22

**Authors:** Isabela Mayá Wayhs Silva, Milena Atique Tacla, Erlane Marques Ribeiro, Elaine Lustosa-Mendes, Agnes Cristina Fett-Conte, Têmis Maria Félix, Ana Carolina Xavier, Isabella Lopes Monlleó, Vera Lúcia Gil-da-Silva-Lopes

**Affiliations:** a Universidade Estadual de Campinas (Unicamp), Departamento de Medicina Translacional, Área de Genética Médica e Medicina Genômica, Campinas, SP, Brazil; b Hospital Infantil Albert Sabin (HIAS), Serviço de Genética Médica, Fortaleza, CE, Brazil; c Centro de Atendimento Integral ao Fissurado Labiopalatal (CAIF-AFISSUR), Curitiba, PR, Brazil; d Faculdade de Medicina de São José do Rio Preto (FAMERP/FUNFARME), Departamento de Biologia Molecular, São José do Rio Preto, SP, Brazil; e Hospital de Clínicas de Porto Alegre (HCPA), Serviço de Genética Médica, Porto Alegre, RS, Brazil; f Centro de Pesquisa e Reabilitação de Lesões Lábio-Palatais (CRRLPL), Joinville, SC, Brazil; g Faculdade de Medicina do Hospital Universitário da Universidade Federal de Alagoas (UFAL), Serviço de Genética Clínica, Maceió, AL, Brazil

**Keywords:** Orofacial cleft, Genetic services, Public health, Cleft lip, Cleft palate

## Abstract

**Objective:**

The current study delves into the accessibility of genetic evaluations for individuals with orofacial clefts (OC), comparing data between genetics and treatment centers across Brazil.

**Methods:**

This cross-sectional retrospective study analyzed primary data from 1463 OC individuals registered in the Brazilian Database of Craniofacial Anomalies (BDCA) between 2008 and 2018 without age or sex selection. Diagnostic exam results stemming from research projects until 2023 were considered.

**Results:**

Of the 1463 individuals with typical OC, 987 were non-syndromic, 462 were syndromic (SOC), 10 presented atypical forms, and three were not specified OC cases. The average age for accessing laboratory diagnosis was 8.5 years among SOC individuals. Notably, more SOC cases were registered in genetics centers than treatment and rehabilitation centers (37.1 % vs. 29 %, *p* = 0.0015). Those originating from genetics centers accessed diagnosis at an average age of 7.3 years, while those from treatment and rehabilitation centers experienced delays with an average age of 10.7 years (*p* = 0.0581).

**Conclusions:**

Irrespective of the center of origin, the data highlight delayed diagnosis and challenges in accessing genetic tests for the syndromic group. Given the widespread reliance on the public health system by most of the Brazilian population, disseminating this data can significantly contribute to shaping an informed perspective on healthcare access. These insights can improve public policies tailored to the unique needs of individuals with OC.

## Introduction

Orofacial clefts (OC), impacting approximately 4.24/10.000 live births in Brazil,^1^ present considerable challenges to affected individuals, compromising aesthetics and functional aspects of life. The complex etiology of OC involves isolated cases influenced by genetic and environmental factors, while syndromic cases typically arise from Mendelian variants, chromosomal alterations and imbalances, and teratogenesis;^2^ unknown causes still represent a significant part of these cases.

The World Health Organization emphasizes a holistic approach to OC cleft care, necessitating collaboration among different specialties, which includes medical genetic services.^3^ In Brazil, public hospitals play a pivotal role in cleft lip and palate correction and rehabilitation, with 30 accredited centers nationwide.^4^ However, it is notable that medical geneticists are not mandatory within them.^5^

Individuals with OC often undergo genetic evaluation and testing at universities or specialized rare disease centers in Brazil. The National Policy for Comprehensive Care for People with Rare Diseases (PNAIPDR), initiated in 2014, advocates for integral and coordinated care within the Brazilian public health system.^6^ Unfortunately, financial constraints, outdated resource allocations, and a scarcity of reference centers in certain states contribute to a substantial backlog of patients awaiting genetic testing.^7^

In 2003, Brazil's Craniofacial Project (BCFP) emerged at Universidade Estadual de Campinas (Unicamp), focusing on individuals with craniofacial anomalies (CFA) and operating on three fronts—health care, genetics of OC and specific conditions, and education. The BCFP, with 12 voluntary participating centers, has significantly contributed to molecular diagnosis, genetic counseling, and insights informing public policies.^8^, ^9^, ^10^, ^11^, ^12^, ^13^ All patient data is voluntarily recorded at the Brazilian Database of Craniofacial Anomalies (BDCA).

In the complex landscape of genetic access for individuals with OC in Brazil, this study seeks to assess the accessibility of diagnostic services for those with these conditions. By comprehensively understanding the existing scenario, the research aims to provide insights to guide potential improvements in genetic services and enhance overall orofacial cleft care in Brazil.

## Methodology

This is a descriptive cross-sectional study focused on analyzing primary data obtained from 1463 individuals with OC. Data were collected during the period spanning from 2008 to 2018. Inclusion criteria encompassed registration with the BDCA, the presence of an orofacial cleft, and comprehensive records of clinical information. There were no age restrictions or sex specifications for inclusion in the study. Exclusion criteria included incomplete/inconsistent data records.

The results of genetic tests carried out until 2023 were included. All data originated from the BDCA and were cross-referenced with the BCFP.

The participating centers within the BCFP were categorized into genetic and rehabilitation/treatment centers; all had genetic evaluations. From the nine centers participating in this study, four were classified as genetic centers and three as rehabilitation/treatment. Two centers could not be classified in any of these categories, and therefore, patients from both were not counted for comparative purposes between genetics and rehabilitation/treatment centers.

The collected data were categorized into sociodemographics, clinics, and information about access to diagnostics. Most diagnostic examinations were conducted within the research framework of BCFP at Unicamp, utilizing methods such as Fluorescence *in situ* hybridization (FISH), Multiplex ligation-dependent probe amplification (MLPA), Chromosomal microarray analysis (CMA), and whole exome sequencing. The only exception is karyotyping, which may have occurred within the Brazilian public or private health system. The age of access to diagnosis corresponded to the date of registration with the BBAC, capturing sociodemographic and clinical data. Individuals who were diagnosed under one year of age had their age at diagnosis changed to zero. Sociodemographic data was not complete for all individuals participating in the study. Therefore, some information has a total number of individuals lower than the total sample.

This study was approved by the Ethics Committee Board of the Universidade Estadual de Campinas (Unicamp), CAAE: 35316314.9.1001.5404 and 5020018.8.0000.5404 and in all participant centers.

All participants or their legal guardians signed the informed consent form.

### Statistical analysis

Descriptive analysis was performed for categorical and numerical variables. When necessary, the Chi-square test, Fisherʼs exact test, or the Mann–Whitney test was applied to compare proportions and groups. The significance level adopted for statistical tests was *p* **<** 0.005.

## Results

### Sociodemographic data of the casuistry

In total, 1463 individuals with OC were included in this study. The majority were male, aged under six, and from the Northeast. Their sociodemographic aspects are described in Table 1.

**Table 1:** Sociodemographic characterization of the casuistry.

**Characteristics**	***n*=1463**	
**Sex**	**F (%)**	**M (%)**
	**664 (45.4)**	**798 (54.6)**
**Age** (in years)	**Mean (s.d.)** 5.14 (7.95)	
	**Median** 1.20	
**Region of residence**	**Total (%)**	
Northeast	820 (56.0)	
Southeast	152 (10.4)	
South	491 (33.6)	

n, number of participants; F, female; M, male; s.d., standard deviation.

### Orofacial clefts classification

The OC observed in this study were classified as typical non-syndromic (NSOC), typical syndromic (SOC), atypical, or not specified. Subsequently, these were classified according to their topographic location as cleft lip, lip and palate, cleft palate, bifid uvula, or atypical OC (Table S1, Supplementary material).

### Orofacial clefts diagnosis

Usually, NSOC diagnosis is essentially made by dysmorphological evaluation and genetic tests are not necessary. A specific diagnosis for atypical and not specified OC individuals was unavailable at BDCA. Data regarding access to diagnosis among the 462 individuals with SOC are described in Table 2.

**Table 2:** Data regarding diagnosis in syndromic orofacial clefts.

**With diagnosis**		***n*** = 132	
		**Laboratorial**	**Clinical**
		68 (51.5 %)	64 (48.5 %)
**Age at diagnosis** (in years)	**Laboratorial**	**Mean (s.d.)**	**Median**
		8.5 (8.1)	7.3
	**Clinical**	**Mean (s.d.)**	**Median**
		4.4 (6)	2.8
	**General**	**Mean (s.d.)**	**Median**
		6.5 (7.4)	4.1

n, number of participants; s.d., standard deviation.

### Genetic centers versus treatment/rehabilitation centers

In total, it was possible to classify the center of origin of 1343 individuals. Among these, 491 (36.6 %) came from genetics centers and 852 (63.4 %) from treatment/rehabilitation. The proportion of the different types of OC according to their classification concerning the center of origin can be seen in Table 3.

**Table 3:** Proportion of orofacial cleft classification according to center of origin.

**Classification**	***n*** = 1343		
	**Genetic centers**	**Tratment/ rehabilitation centers**	
	**Total (%)**		
Non-syndromic	301 (61.3)	**600 (70.4)**	*p* = 0.0015^a^
Syndromic	**182 (37.1)**	247 (29)	
Atypical	6 (1.2)	4 (0.5)	
Not specified	2 (0.4)	1 (0.1)	

n, number of participants.

^a^Based on Fisher's exact test.

The proportion of individuals with syndromic orofacial cleft who were diagnosed clinically and by genetic testing concerning the centers of origin are described in Table 4.

**Table 4:** Center of origin versus type and age of diagnosis in SOC.

**Total (%)**	***n*** = 124				*p* = 0.0177^a^
	**Genetic centers**		**Treatment/ rehabilitation centers**		
	Laboratorial	Clinical	Laboratorial	Clinical	
	**39 (65)**	21 (35)	28 (43.8)	**36 (56.3)**	
Age of diagnosis	**Mean (s.d.)**	**Mean (s.d.)**	**Mean (s.d.)**	**Mean (s.d.)**	*p* = 0.00581^b^
	7.3 (7.6)	3.4 (4)	10.7 (8.4)	5.2 (7.1)	

*n*, number of participants; SOC, syndromic orofacial cleft.

^a^Based on Chi-square test.

^b^Based on Mann–Whitney test.

## Discussion

This study reflects the BCFP/BDCA strategy to address the gap in access to genetic evaluation and diagnostic testing for OC among its participant centers. Therefore, the geographical distribution of individuals, type, or topographic location of OC recorded does not reflect an epidemiological pattern, nor the absence of OC diagnosis in the North and Central West regions. The composition of the sample group aligns with the prevailing distribution observed in the literature concerning individuals with SOC and NSOC.^14^

Of the 462 individuals with SOC, 28.6 % received a diagnosis. Although a notable proportion (51.5 %) received a molecular diagnosis, the mean age of molecular diagnosis was 8.5 years. In contrast, access to clinical diagnosis occurred approximately two times earlier for 48.5 % of individuals who obtained it.

This temporal contrast between access to clinical versus molecular diagnosis underscores the challenges of accessing genetic tests in Brazil. The limitations within the public health system, including restricted funding for genetic testing, a scarcity of genetic services, and delays in the arrival of patients at the reference service, contribute to this disparity.^7^^,^^13^^,^^15^, ^16^, ^17^, ^18^ Conversely, while offering genetic testing, private healthcare still needs to be financially accessible for most of the Brazilian population.^19^

Acting voluntarily and with resources from research funding agencies, the BCFP incorporates a small group of patients, as they are included in research projects. These limitations do not allow all individuals referred for clinical follow-up at BCFP to access genetic testing.^12^

In this context, revising the standards for OC public service accreditation with the inclusion of medical genetics (for evaluation and genetic tests) or establishing consistent interaction with the PNAIPDR would benefit the Brazilian public OC care policy. This strategic measure aims to enhance and speed up identifying potential diagnoses, ensuring swift referrals to more targeted genetic tests.

Regarding the disproportion between syndromic individuals coming from genetics centers versus treatment/rehabilitation centers, it is essential to mention that, most likely, when there is a genetic center close to where the person with SOC resides, there is a greater probability that they will be referred further quickly to these centers to define the diagnosis. When there are only treatment/rehabilitation centers, the referral of people with SOC can be directed, preferably, to centers specializing in rare genetic diseases.

For instance, within the cohort of individuals with SOC originating from rehabilitation/ treatment centers, the average age of molecular diagnosis obtained was 10.7 years, contrasting with 7.3 years in genetic centers. Even so, it is noteworthy that all genetic tests were carried out within the BCFP. Ensuring timely access to genetic services is crucial in streamlining genetic counseling and providing comprehensive clinical follow-up for patients.^20^ These data underscore the indispensable role of geneticists in this domain.

The results show that the age at which individuals with syndromic orofacial cleft access diagnostic tests is late but similar to what is observed for rare diseases in Brazil.^21^ The current scenario reinforces a critical need for interventions to improve the accessibility of genetic testing, particularly within the public health system. This study underscores the importance of advocating for enhanced resources, increased distribution of genetic services, and reduced financial barriers to ensure comprehensive care for this population. Specifically for oral clefts, targeted public policies involving genetic evaluation and diagnosis can increase health care.

### Data availability of data and statement

The datasets generated and analyzed during the current study are not publicly available because they contain personal information from patients.

### Funding statement

This work received funding from the National Council for Scientific and Technological Development—CNPq [#140354/2020-4; #309782/2020-1 and 304684/2023-6]. The Postgraduate Program in Medical Sciences is supported by Coordination for the Improvement of Higher Education Personnel—CAPES [# 001].

## Conflicts of interest

The authors declare no conflict of interest.

## Appendix Supplementary materials

Image, application 1

## Footnotes

Supplementary material associated with this article can be found in the online version at doi:10.1016/j.jped.2024.07.002.
