# Supplementary material for: Access to genetic evaluation of 1463 individuals with orofacial cleft in Brazil
Source: J Pediatr (Rio J). 2024 Jul 22;100(6):604–8. doi: 10.1016/j.jped.2024.07.002 (PMC11662741; doi:10.1016/j.jped.2024.07.002)
Supplement: Supplementary file 1 [file mmc1.docx]

**JPED-D-24-00149 – Supplementary Material**

**Appendix**

Table S1 Orofacial cleft classification and topography.

| **Classification** | **n = 1463** | | |
| --- | --- | --- | --- |
|  | **Total (%)** | **Topographic classification** | **Total (%)** |
| Non-syndromic | 987 (67.5) | **Cleft lip** | 225 (22.8) |
|  |  | **Cleft lip and palate** | 495 (50.2) |
|  |  | **Cleft palate** | 264 (26.7) |
|  |  | **Bifid uvula** | 3 (0.3) |
| Syndromic | 462 (31.6) | **Cleft lip** | 37 (8) |
|  |  | **Cleft lip and palate** | 187 (40.5) |
|  |  | **Cleft palate** | 221 (47.8) |
|  |  | **Bifid uvula** | 10 (2.2) |
|  |  | **Atypical** | 7 (1.5) |
| Atypical | 10 (0.7) | - | - |
| Not specified | 3 (0.2) | **Cleft lip and palate** | 3 (100) |

n, number of participants.
